# SmoPSI: Analysis and Prediction of Small Molecule Binding Sites Based on Protein Sequence Information

**DOI:** 10.1155/2019/1926156

**Published:** 2019-11-13

**Authors:** Wei Wang, Keliang Li, Hehe Lv, Hongjun Zhang, Shixun Wang, Junwei Huang

**Affiliations:** ^1^Department of Computer Science and Technology, College of Computer and Information Engineering, Henan Normal University, 453007 Xinxiang, Henan Province, China; ^2^Laboratory of Computation Intelligence and Information Processing, Engineering Technology Research Center for Computing Intelligence and Data Mining, 453007 Xinxiang, Henan Province, China; ^3^School of Aviation Engineering, Anyang University, 455000 Anyang, Henan Province, China

## Abstract

The analysis and prediction of small molecule binding sites is very important for drug discovery and drug design. The traditional experimental methods for detecting small molecule binding sites are usually expensive and time consuming, and the tools for single species small molecule research are equally inefficient. In recent years, some algorithms for predicting binding sites of protein-small molecules have been developed based on the geometric and sequence characteristics of proteins. In this paper, we have proposed SmoPSI, a classification model based on the XGBoost algorithm for predicting the binding sites of small molecules, using protein sequence information. The model achieved better results with an AUC of 0.918 and an ACC of 0.913. The experimental results demonstrate that our method achieves high performances and outperforms many existing predictors. In addition, we also analyzed the binding residues and nonbinding residues and finally found the PSSM; hydrophilicity, hydrophobicity, charge, and hydrogen bonding have obviously different effects on the binding-site predictions.

## 1. Introduction

Proteins perform the biological functions through interactions with other molecules. In most cellular processes, proteins interact with small molecules to perform their biological functions. Therefore, the prediction of protein-small molecule binding sites is of great significance for understanding and exploring the function of proteins [1–6]. With the continuous development of molecular pathology, people have a deeper understanding of the molecular mechanism of disease occurrence and drug efficacy. The study of the interaction between drug molecules and protein targets is the basis of the drug design process. In recent years, many calculation methods have been proposed for the problem of drug molecule and protein binding sites. These methods are fast and inexpensive compared to traditional biochemical experiments. The identification methods for binding sites were mainly classified into the following categories. The purely geometric-based approach follows the assumption that the protein-small molecule binding site is usually located in the gap of the protein surface or the pores of the protein. For example, the CASTp algorithm can locate and measure the 3-dimensional structure of protein pockets [7]. Fpocket is a protein surface pocket and space detection package based on Voronoi tessellation and alpha spheres. It enables fast and efficient protein pocket detection and efficient pocket descriptor extraction [8]. PocketPicker is a grid-based automation technology for predicting protein binding pockets and specifying the shape and burial of potential binding sites [9]. The SITEHOUND algorithm identifies potential ligand-binding sites and regions characterized by favorable nonbonded interactions with a chemical probe [10]. Based on sequence data methods, protein sequences can be used to identify binding sites for ligand protein molecules. ATPsite combines secondary structure, solvent accessibility, and dihedral angles based on evolutionary information to construct the SVM classifier to predict the ATP binding residue [11]. WideDTA is a deep learning-based prediction model that employs protein sequence to drug-target binding affinity [12]. TargetS extracts three characteristics of evolutionary information, secondary structure, and ligand-specific binding propensity features. Based on these features, an Adaboost classifier scheme is proposed [13]. Yu et al. also proposed a classification scheme based on the PSSM and secondary structure of protein sequences [14–16]. In order to predict binding residues, TargetATPsite proposes a novel image sparse representation technique for encoding PSSMs of sequence data [17]. In addition, Q-SiteFinder utilizes the interaction energy between the protein and the van der Waals probe of the ligand to locate favorable binding sites [18]. Dai proposed a solution that not only uses PSSM features based on sequence data but also combines methods based on geometric cavity recognition.

Inspired by the previous work, in our study, we present a new SmoPSI method for predicting the binding sites of small molecules to protein targets based on protein sequence. The SmoPSI model was obtained by training the binding residue and nonbinding residue using the XGboost classifier which is an integrated learning algorithm based on gradient boosting. The SmoPSI method can predict binding domain residues based on the physicochemical properties and evolutionary information of extracting protein sequence residues. The PSSM of protein sequences has been used to predict binding sites, because whether the residues in the sequence are mutated is determined by many factors in the evolution process, and these factors also affect the binding of proteins to other protein-small molecules [19, 20]. The physicochemical properties of the binding domain residues and the nonbinding domain residues are also different. For example, the binding domain of protein and DNA molecules exhibits a higher single negative charge characteristic [21–25]. Therefore, we analyzed the hydrophobic, hydrophilic, charge, and hydrogen bond properties of the binding domain residues and nonbinding domain residues on the protein sequence. We also present the type information of the amino acid residues in the feature matrix in the form of a one-hot code and analyze the distribution of different residue types between the binding residues and the nonbinding residues. Based on the above features, we used the XGBoost algorithm for classification and prediction. The full name of XGBoost is eXtreme Gradient Boosting, which is an integrated learning algorithm based on gradient boosting [26]. Finally, we trained and verified 14 classes of protein-small molecules. The model SmoPSI achieved better results with an AUC of 0.918 and an ACC of 0.913 and obtained a more significant classification effect.

## 2. Datasets and Method

### 2.1. Datasets

In the experiment, we obtained the dataset of protein-small molecules from the SC-PDB database (http://bioinfopharma.ustrasbg.fr/scPDB/) [27]. As of August 2018, 16034 entries, 4782 proteins and 6326 ligands, have been released on the SC-PDB website. We removed redundant data using the PISCES program (http://dunbrack.fccc.edu/Guoli/PISCES.php) after downloading all of the protein-small molecules. After removal of redundancy, 5090 protein-small molecules were screened with sequence identity less than 30% and a minimum chain length of 40 amino acid residues [28]. The realization of protein function needs to recognize the binding residue of the ligand molecule in the protein by binding to the ligand, which will provide important help for protein function research and drug development. Here, if the distance between the central atom of residues and any one of the ligand molecules is less than 3.5 Å, this residue is a binding residue. Conversely, it is a nonbinding residue. Finally, we chose 14 types of protein-small molecules that are large enough to be useful for testing, as shown in Table 1. The number of protein sequences to each protein-small molecule and the number of bound residues and nonbinding residues in the sequence are shown, respectively. However, because of the large difference in the number of binding residues and nonbinding residues, there is a significant data imbalance problem in the dataset. So, in the course of the experiment, we used a downsampled method to repeatedly down sample the nonbinding residues during the training. We found through experiments the downsampled method can solve the problem of data imbalance.

### 2.2. PSSM

The conservatism in the evolution of residues at the various positions of a protein sequence can be reflected by the evolutionary information of the protein. The evolutionary information of proteins is obtained by multisequence comparison of homologous sequences. Because the evolutionary information of proteins base on all members of the homologous family, it can fully respond to distant homologous relationships [29]. PSI-BLAST is a position-specific iterative basic local alignment search tool. We used it to scan the NCBI database with an *e* value of 0.001 and a number of iterations of 3, resulting in a *L* × 20 position-specific scoring matrix (PSSM) of the protein sequence (*L* is the length of the sequence).

### 2.3. Physicochemical Properties

Hydrophilicity characterizes the amount of protein residues that are compatible with water, and hydrophobicity indicates the ability to repel water. Hopp gave a hydrophobicity scale for each of the 20 amino acids with a fixed value (AAindex ID: HOPT810101). Patrick gave a hydrophilicity scale for each of the 20 amino acids with a fixed value (AAindex ID: HOPT810101). It is generally believed that amino acids having hydrophobicity tend to be oriented toward the interior of the protein structure, while amino acids having hydrophilicity tend to face the surface in the three-dimensional structure of the protein [30]. Therefore, hydrophilic amino acids have a greater probability of interacting with small molecules. Such features help distinguish between the binding residues and the nonbinding residues. The electrostatic charge of the binding region of a protein to a small molecule is one of the most influential properties of protein-ligand interactions, which has been confirmed by many studies [31]. Electrostatic complementation contributes to the nonspecific binding of proteins to small molecules [32]. Among the 20 standard amino acids, Arg, His, and Lys are positively charged, and Asp and Glu are negatively charged. We believe that the electrostatic charge value of a protein residue is helpful in distinguishing between binding residues and nonbinding residues.

Hydrogen bonds are a kind of interaction force that is slightly stronger than the intermolecular force and slightly weaker than the covalent bond and the ionic bond. The hydrogen bond plays an important role in the interaction between the protein and the small molecules [33]. The interaction of a protein with small molecules may depend primarily on the hydrogen bonding ability of the atoms in the side chain of the amino acid. Therefore, the number of hydrogen bonds contained in an amino acid is also one of its important physical and chemical properties. The hydrogen bonding properties of amino acids can be obtained from the AAindex database (AAindex ID: FAUJ880109) of the 20 amino acids, and 11 amino acids can form hydrogen bonds through their side chains.

### 2.4. Sliding Window Sampling

**Figure 1:**
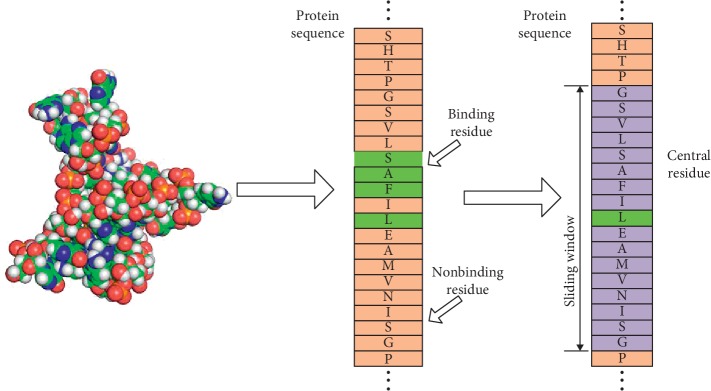
The sliding window sampling of protein sequences. A residue on the protein sequence is used as the center point, with (*w* − 1)/2 as the boundary, and then the extracted matrix is the feature of the central residue. The features are extracted matrix of the entire protein sequence in the sliding.

For predicting the binding residues of proteins to small molecules, extracting information from sequences and constructing feature vectors is a very critical step. Since the data used in the experiment are sequence data, the binding residues and the nonbinding residues are continuously distributed, so we sought to create a sliding sampling window on the protein sequence. As shown in Figure 1, the sampling window contains the binding residues and (*w* − 1)/2 adjacent residues on both sides of the residue. We used eight different initial values as an alternative to the window length *w* [33]. If the residue in the center of the window is a binding residue, then we consider the matrix sampled by the window to be a positive set. A matrix sampled by a window is considered to be a negative set if the residue at the center of the window is a nonbinding residue. We believed that the binding residues are also affected by the environment surrounding the binding site residues.

### 2.5. Classification Model

Tree boosting is an efficient and widely used machine learning method [26]. We use an extensible end-to-end tree boosting system XGBoost, which is widely used by scientists to achieve the most advanced results in many machine learning challenges. Gradient boosting is an improvement on the basis of boosting. The idea of the algorithm is to continuously reduce the residuals and further reduce the residual of the previous model in the gradient direction to obtain a new model. XGBoost is just an improved version of the Gradient Boosting algorithm. XGBoost generates a second-order Taylor expansion for the loss function and obtains the optimal solution for the regular term outside the loss function, making full use of the parallel computing advantages of the multicore CPU to improve the accuracy and speed.

### 2.6. Evaluation Measurements

The following evaluation criteria were used in our experiments: the overall prediction accuracy (ACC), F1, precision, recall, and the area under the ROC (receiver operating characteristic) curve (AUC). The performance measures are defined as

(1) $$\begin{array}{c} \text{ACC}=\;\frac{\left(\text{TP}+\text{TN}\right)}{\left(\text{TP}+\text{FN}+\text{TN}+\text{FP}\right)},\\ \text{precision}=\frac{\text{TP}}{\left(\text{TP}+\text{FP}\right)},\\ \text{recall}=\frac{\text{TP}}{\left(\text{TP}+\text{FN}\right)},\\ \text{F}1=\;\frac{2\;*\;\left(\text{precision }*\text{ recall}\right)}{\left(\text{precision}+\text{recall}\right)}. \end{array}$$

These metrics can be measured by the numbers of true positives (TP), false positives (FP), true negatives (TN), and false negatives (FN) for each classifier, where TP is the number of proteins correctly predicted as binding sites, FP is the number of proteins incorrectly predicted as binding sites, TN is the number of proteins correctly predicted as no binding sites, and FN is the number of proteins incorrectly predicted as no binding sites. The area under the receiver operating characteristic (AUC) is an evaluation method for a predictor in a binary classification system.

## 3. Results and Discussion

### 3.1. Distribution of Amino Acid Classes

**Figure 2:**
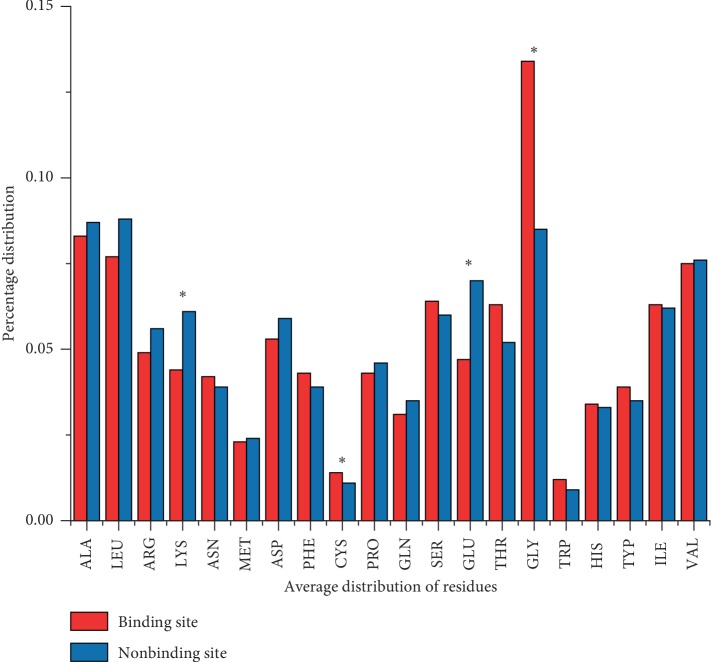
Distribution of 20 amino acids between binding residues and nonbinding residues in the protein sequences.

We collected the molecular sequence of 6592 protein-small molecules and analyzed the type and distribution ratio of binding and nonbinding residues in all protein sequence data, as shown in Figure 2. For nonbinding residues, Ala, Leu, Gly, Val, and Glu are more distributed. In addition, we can also find that the distribution differences of Gly, Glu, Cys, and Lys are statistically significant for the binding residues and nonbinding residues. Differences in the distribution of these classes of amino acids may be related to the function of binding residues to nonbinding residues.

### 3.2. Analysis of Physicochemical Properties of Protein Binding Regions

**Figure 3:**
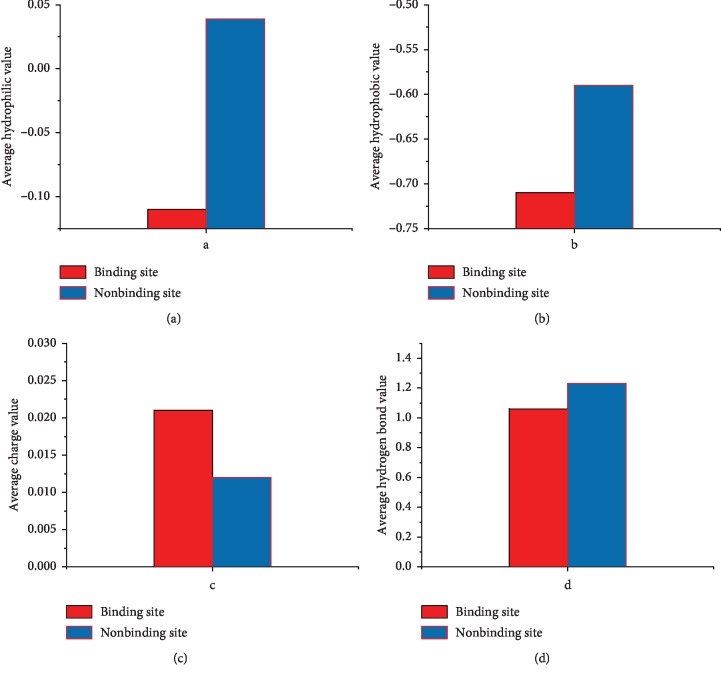
Distribution of four physicochemical properties of binding domain residues and nonbinding domain residues in the protein sequences.

In addition to analyzing the distribution of each type of amino acid, we calculated the hydrophilicity, hydrophobicity, electrostatic charge, and hydrogen bond distribution of the protein binding domain and the nonbinding domain, as shown in Figures 3(a) and 3(b). The hydrophilicity and hydrophobicity are significantly different between the bound and unbound regions. The likely cause is because the binding domain residues are mostly distributed on the surface of the protein, the binding domain residues are more likely to contact with water molecules [30]. The hydrophilicity value and the hydrophobicity value represent the tendency to be hydrophilic and hydrophobic. As shown in Figure 3(c), we have found that the binding region exhibits a hydrophilic character and the nonbinding region exhibits a hydrophobic character. However, the electrostatic distribution of the nonbonded regions is still smaller than the bonded regions. Then, we think that electrostatic complementation helps the binding of proteins to small molecules [34, 35]. Although previous studies have found that hydrogen bond plays an important role in ligand and protein binding, we have no statistically significant difference in the distribution of hydrogen bonds between binding and unbinding regions [36, 37]. Based on the aforementioned findings, hydrophilicity, hydrophobicity, and electrostatic charge of amino acids help us to construct a classification model for the binding residues and nonbinding residues.

### 3.3. Classification Performance

We evaluated our method on 14 molecular datasets of protein-small molecules. The 14 kinds of small molecules are ACO, ADP, ANP, ATP, COA, FAD, FMN, GDP, GNP, NAD, NAP, NDP, SAH, and SAM. The details of the dataset corresponding to each type of small molecule are shown in Table 1, including three types of nucleotides (ATP, ADP, and GDP). The receptor for atrial natriuretic peptide (ANP) is a type-I transmembrane protein containing an extracellular ligand-binding domain, a single transmembrane sequence, an intracellular kinase-homologous domain, and a guanylate cyclase (GCase) domain [38]. ADP is the cognate ligand for the orphan G protein-coupled receptor [39].

### 3.4. Determination of the Length of the Sampling Window

**Figure 4:**
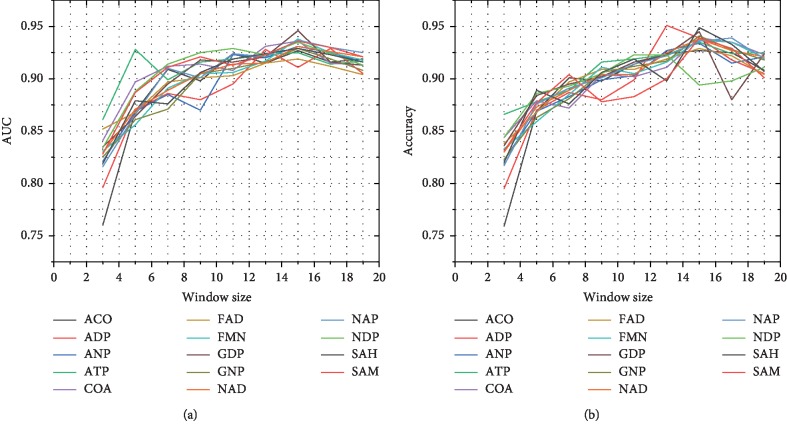
The analysis between the sampling window *w* and predication performances. (a) With the length of the sampling window increasing, the AUC values of 14 protein-small molecules continue to increase. When *w*=15, the AUC values of all protein-small molecules reach a peak and then decrease. (b) With the length of the sampling window increasing, the ACC values of 14 protein-small molecules increase continuously. When *w*=15, ACC values of all protein-small molecules reach a peak and then decrease.

The width of the sampling window in data processing directly determines the quality of the dataset and the experimental effect of the classification. Therefore, we tested the alternative 8 window lengths on the 14 molecular sets. We used the downsampling method for the phenomenon that the number of positive and negative datasets is different and then carried out 10-fold cross-validation. We selected two indicators AUC and ACC as the criteria for window width selection. The relationship between AUC and window width is shown in Figure 4(a). When the window is scaled up to 3, the AUC shows an upward trend. If *w*=15, the AUC values of all types of datasets reach maximum values. Then, the value of AUC begins to decrease. The docking on the protein surface is not only related to the residue itself but also related to the surrounding environment. When the window length is small, the sampled digital matrix does not effectively represent the surrounding features. Therefore, values of AUC and ACC are small. Due to the small size of small molecules, the number of bound residues in one sequence is not large. When the sampling length is too long, the sampled digital matrix will contain a lot of interference information, resulting in a small value of AUC and ACC too. The same result can also be observed in Figure 4(b). Because the result of ACC reaches a maximum at *w*=15, we choose the window width *w* to be 15 residues.

### 3.5. Significance Testing of the Features

**Figure 5:**
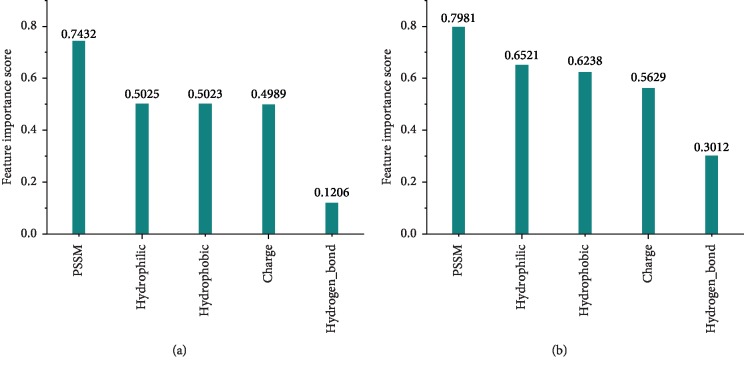
The feature significance scores are calculated using the mean decrease accuracy and random forest-based feature importance scoring system. (a) Mean decrease accuracy. (b) Random forest-based feature importance scoring system.

In the previous discussion, we introduced and used five features of proteins: PSSM, hydrophilic, hydrophobic, electrostatic charge, and hydrogen bonding. To analyze the significance of different features, we used the mean decrease accuracy and random forest-based feature importance scoring system to measure the effect of each feature on the predictive model. The basic idea of the algorithm is to change a certain feature value to a random number and observe how much the accuracy of the model is reduced. For unimportant features, this method has little effect on the accuracy of the model, but it will greatly reduce the accuracy of the model for important features, as shown in Figure 5. The PSSM has the highest score for the features, followed by hydrophilicity, hydrophobicity, and charge. Hydrogen bonds have the least impact on the classification model. The above results are also consistent with the previous analysis of the protein binding region.

### 3.6. Performance of the Classification Model

We constructed these feature vectors with amino acid classes, PSSMs, hydrophilicity, hydrophobicity, and charge, while the length of the window is set at the length of 15 residues. Then, 14 kinds of protein-small molecules were classified and predicted. The results are shown in Table 2. We found that the AUC and ACC results for the ATP type of protein-small molecules are highest at 0.935 and 0.927, respectively. The average AUC and ACC of all 14 species of protein-small molecules are 0.918 and 0.913, respectively. The strategy of SmoPSI is useful to improve the performance of prediction.

### 3.7. Comparison with Existing Predictors

Our method is compared with EC-RUS and TargetS, and results on the training sets are listed in Table 3. The recall values of our method are 0.934, 0.940, and 0.940 on ADP, ATP, and GDP, respectively. TargetS [13] achieves recall values of 0.561, 0.484, and 0.639 on ADP, ATP, and GDP, respectively. EC-RUS [40] achieves recall values of 0.561, 0.484, and 0.639 on ADP, ATP, and GDP, respectively. Obviously, the performance of the proposed method is better than that of TargetS and EC-RUS. In addition, our method achieves an average AUC value of 0.933, which is better than the average AUC values of TargetS and EC-RUS.

## 4. Conclusion

In previous studies of sequence-based binding site prediction, various methods did not combine PSSM features with the physicochemical properties of proteins. A different method was used in our experiments. First, the differences between the four types of physicochemical properties of hydrophilicity, hydrophobicity, electrostatic charge, and hydrogen bonding in the bound and unbound regions were analyzed. We have found that the residues in the binding region of the small molecule exhibit a hydrophilic character, and the charge characteristics of the binding region are also stronger than those of the nonbinding region. The distribution of hydrogen bonds in the two regions is never significantly greater than others, which can also be observed from the results of the feature significant analysis. At the same time, the distribution of different kinds of amino acids was also analyzed. We concluded that the distribution of five residues such as ALA and LEU was more than that of other amino acids. The distribution of the four residues of GLY, GLU, CYS, and LYS in the binding region and the nonbinding region is significant. We constructed the feature vectors using the aforementioned features and the PSSM. The SmoPSI model was constructed using the XGBoost algorithm and tested on 15 distinct types of protein-small molecules. The average AUC value and ACC value obtained by the 10-fold cross-validation were 0.918 and 0.913, respectively. The SmoPSI method can help to understand the binding mechanism between proteins and small molecules and explore the characteristics of small molecules combined with proteins.

## Figures and Tables

**Table 1:** Detailed compositions of the 14 types of small molecules for binding sites.

Small molecular type	Hetnam	Formula	Number of sequences	Number of binding domain residues	Number of nonbinding domain residues
ACO	Acetyl coenzyme	C_23_ H_38_ N7 O_17_ P_3_ S	167	86	4256
ADP	Adenosine-5′-diphosphate	C_10_ H_15_ N_5_ O_10_ P_2_	807	576	20406
ANP	Phosphoaminophosphonic acid-adenylate ester	C_10_ H_17_ N_6_ O_12_ P_3_	386	223	10073
ATP	Adenosine-5′-triphosphate	C_10_ H_16_ N_5_ O_13_ P_3_	549	574	13700
COA	Coenzyme A	C_21_ H_36_ N_7_ O_16_ P_3_ S	350	245	8855
FAD	Flavin-adenine-dinucleotide	C_27_ H_33_ N_9_ O_15_ P_2_	876	1417	21359
FMN	Flavin mononucleotide Riboflavin monophosphate	C_17_ H_21_ N_4_ O_9_ P	425	552	10498
GDP	Guanosine-5′-diphosphate	C_10_ H_15_ N_5_ O_11_ P_2_	214	294	5270
GNP	Phosphoaminophosphonic acid-guanylate ester	C_10_ H_17_ N_6_ O_13_ P_3_	187	344	4518
NAD	Nicotinamide-adenine-dinucleotide	C_21_ H_27_ N_7_ O_14_ P_2_	1053	1305	26073
NAP	NADP nicotinamide-adenine-dinucleotide phosphate	C_21_ H_28_ N_7_ O_17_ P_3_	529	806	12948
NDP	NADPH dihydro-nicotinamide-adenine-dinucleotide Phosphate	C_21_ H_30_ N_7_ O_17_ P_3_	334	462	8222
SAH	S-adenosyl-L-homocysteine	C_14_ H_20_ N_6_ O_5_ S	465	371	11719
SAM	S-adenosylmethionine	C_15_ H_22_ N_6_ O_5_ S	240	186	6054

**Table 2:** The results of classification prediction of 14 protein-small molecules are given by using the SmoPSI model.

Small molecules	ACC	Precision	Recall	F1	AUC
ACO	0.900	0.886	0.921	0.904	0.891
ADP	0.910	0.891	0.934	0.912	0.927
ANP	0.900	0.863	0.940	0.900	0.897
ATP	0.927	0.914	0.940	0.927	0.935
COA	0.928	0.918	0.940	0.929	0.931
FAD	0.903	0.875	0.932	0.903	0.917
FMN	0.926	0.911	0.940	0.925	0.932
GDP	0.929	0.917	0.940	0.928	0.937
GNP	0.926	0.914	0.940	0.927	0.925
NAD	0.916	0.898	0.937	0.917	0.922
NAP	0.915	0.893	0.940	0.916	0.912
NDP	0.906	0.875	0.940	0.907	0.908
SAH	0.875	0.830	0.930	0.877	0.894
SAM	0.926	0.914	0.940	0.927	0.920
Average	0.913	0.893	0.937	0.914	0.918

**Table 3:** Comparison with existing predictors on training sets of 3 same types of ligands.

Predictor methods	Small molecules	ACC	Recall	AUC
SmoPSI	ADP	0.910	0.934	0.927
	ATP	0.927	0.940	0.935
	GDP	0.929	0.940	0.937
	Average	0.922	0.938	0.933

TargetS	ADP	0.972	0.561	0.907
	ATP	0.962	0.484	0.887
	GDP	0.972	0.639	0.908
	Average	0.969	0.561	0.901

EC-RUS	ADP	0.973	0.622	0.939
	ATP	0.964	0.586	0.912
	GDP	0.976	0.672	0.937
	Average	0.971	0.627	0.929

## Data Availability

The data used to support the findings of this study are available from the corresponding author upon request.
